# Why is glycocholic acid sodium salt better than deoxycholic acid sodium salt for the preparation of mixed micelle injections?

**DOI:** 10.1002/fsn3.1224

**Published:** 2019-09-27

**Authors:** Tao Wang, Liao Shen, Lijun Sun, Yadan Zhang, Haiyan Li, Yongan Wang, Dongqin Quan

**Affiliations:** ^1^ State Key Laboratory of Toxicology and Medical Countermeasure Beijing Institute of Pharmacology and Toxicology Beijing China; ^2^ 309 Hospital of PLA Beijing China

**Keywords:** deoxycholic acid salt, glycocholic acid salt, mixed micelle injection, physiochemical stability, Vitamin K_1_

## Abstract

Classical mixed micelle systems make excellent parenteral drug carriers for lipophilic or poorly soluble drugs, but many formulations details are not fully understood and need further study. Thus, we constructed mixed micelle systems with lecithin and either glycocholic acid sodium salt or deoxycholic acid sodium salt in order to investigate the differences between the bile salts. Vitamin K_1_, a lipid‐soluble drug, was encapsulated in the mixed micelles, and the influence of bile salts on the quality and stability of the mixed micelle systems was analyzed. Both bile salts displayed similar profiles, and the amounts of bile salts used in formulating clear solutions did not differ. Mixed micelle systems formed from glycocholic acid sodium were physically stable at low pH levels (5.5), whereas those formed from deoxycholic acid required higher pH (>8.5). High pH levels hurt active pharmaceutical ingredients that are prone to hydrolytic and oxidative degradation. Hence, when mixed micelle systems formed from deoxycholic acid sodium were sterilized, unexpected chemical unstability occurred. Therefore, we conclude that glycocholic acid sodium salt is more suitable than deoxycholic acid sodium salt for the preparation of mixed micelle injections.

## 1 INTRODUCTION

Classical mixed micelle (MM) systems, composed of natural phospholipids and bile salts, offer a nontoxic, highly biocompatible, and parenterally well‐tolerated drug carrier for lipophilic and poorly soluble drugs. Researchers have attempted various approaches (Amedee‐Manesme, Grüter, & Hanck, 1991; Hammad & Müller, 1998; Mrestani, Behbood, Härtl, & Neubert, 2010; Muranishi, Muranushi, & Sezaki, 1979), but only one product (Konakion^®^ MM) has been approved for the pharmaceutical market; while a few published results have described the influence of lecithin/bile salt ratios and the effect of total concentration on the size and shape of MMs (Krishnadas, Rubinstein, & Önyüksel, 2003; Lichtenberg, Robson, & Dennis, 1983; Shankland, 1970), many other factors are not fully understood. Therefore, more basic studies are required for elucidating the formation and stability of MM preparations.

**Figure 1:**
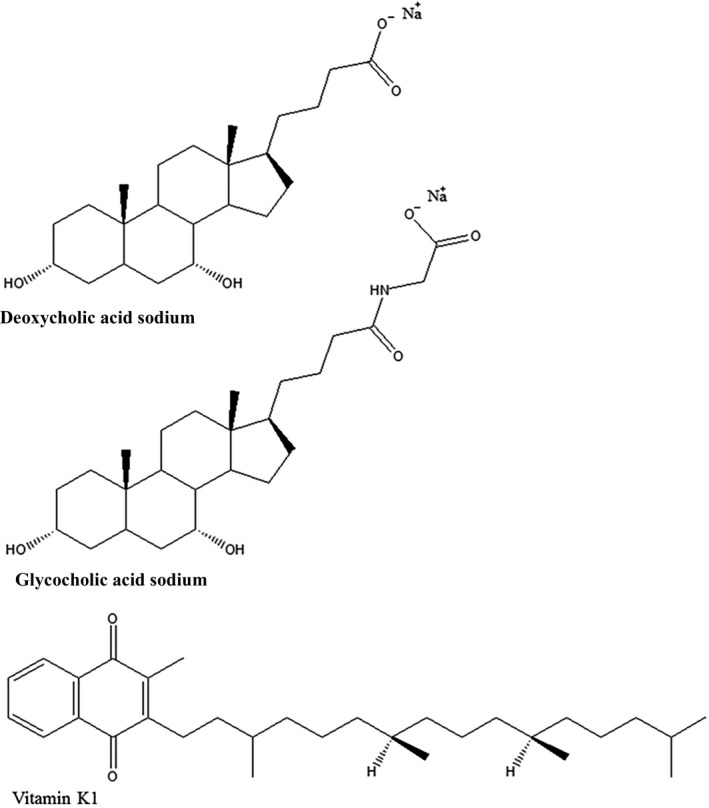
The chemical structures of deoxycholic acid sodium salt, glycocholic acid sodium salt, and vitamin K_1_

Glycocholic acid sodium (GAS) salt and deoxycholic acid sodium (DAS) salt are the most common cholic acid salts in the pharmaceutical industry, especially for the preparation of MMs. Both are water‐soluble anionic surfactants that have the same steroidal backbones (Figure 1). Their physiochemical properties are so similar that their differences are often ignored. However, these slight differences affect the quality of MM injections very strongly in some times. So it is important to know the details to investigate how they differ, we constructed MM systems from lecithin and bile (GAS or DAS) salts. Vitamin K_1_ (Figure 1), a lipid‐soluble drug, was selected as the model drug for encapsulation in the MMs, after which the influence of bile salts on the quality and stability of MM formulations was analyzed.

All MM solutions or dispersions consisted of 1% vitamin K_1_ (Shandong Guangtongbao Pharmaceuticals Co. Ltd.), 4.0 ~ 7.0% phospholipids, and 2.0 ~ 7.0% bile salts in double‐distilled water that was prepared by a film‐forming or evaporation method. The egg phospholipid Lipoid E80 (Lipoid GmbH), as well as GAS or DAS (Sigma‐Aldrich), and vitamin K_1_ were dissolved in ethanol (10 ml). After the solvent was evaporated by a rotary evaporator, pure water was added to obtain a solution of MMs loaded with vitamin K_1_.

**Figure 2:**
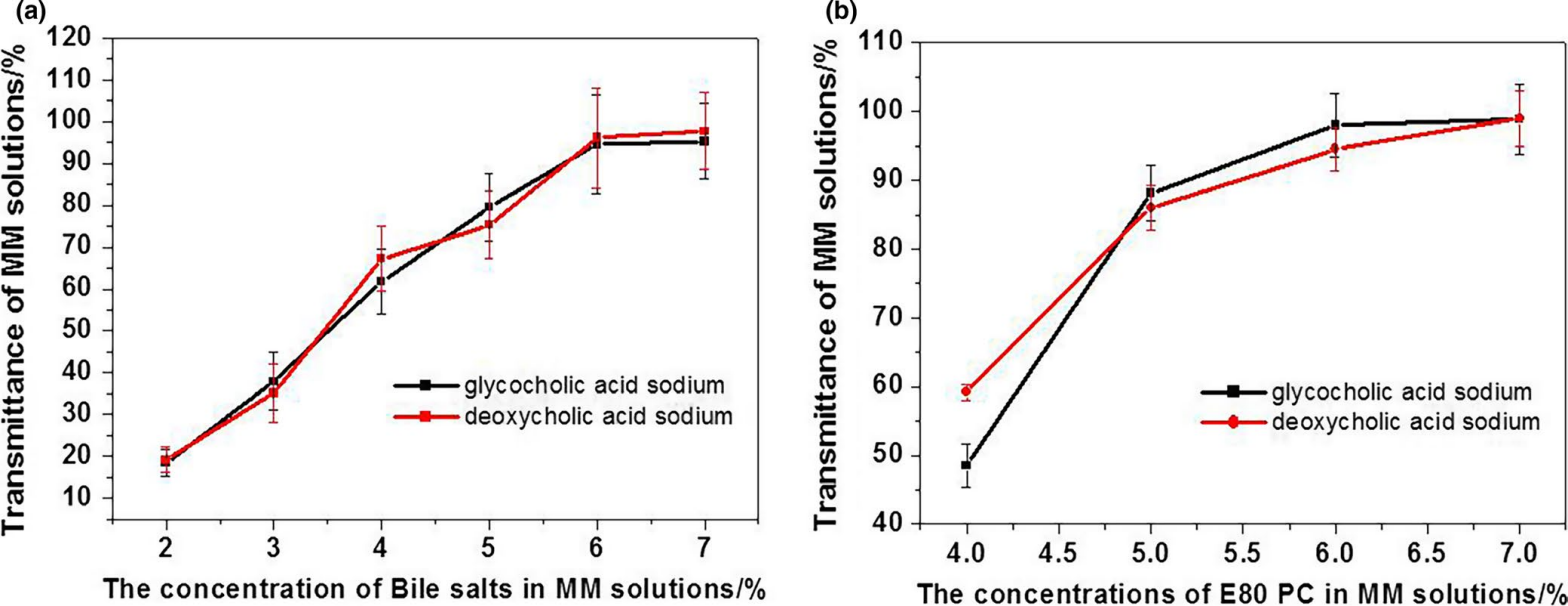
The transmittances of mixed micelle solutions with different concentrations of phospholipid (PC) E80 and bile salts (650 nm; mean ± *SD*, *n* = 3): (a) the concentrations of bile salts ranged from 2.0% to 7.0% while that of PC E80 was constant (6.0%); (b) the concentrations of PC E80 ranged from 4.0% to 7.0% while that of bile salts was constant (6.0%)

For an MM formulation to serve as a good parenteral drug carrier system, it must form an isotropically clear solution. An ultraviolet‐visible spectrophotometer (U‐3010, Hitachi) was used to analyze the transmittance of MM systems at 650 nm in order to elucidate the influence of bile salt or phospholipid (PC) concentrations on the formation of clear solutions in the presence of 1% (m/v) vitamin K_1_. Clear solutions were obtained with high concentrations (>5%) of PC or bile salts (Figure 2): The more bile salts and phospholipids, the more hydrophobic core was available to encapsulate vitamin K_1_. Both GAS and DAS salts exhibited similar abilities to form isotropically clear solutions with the same amounts of PC and vitamin K_1_. Thus, the formation of isotropically clear solutions was dependent on concentration rather than type of bile salt.

**Figure 3:**
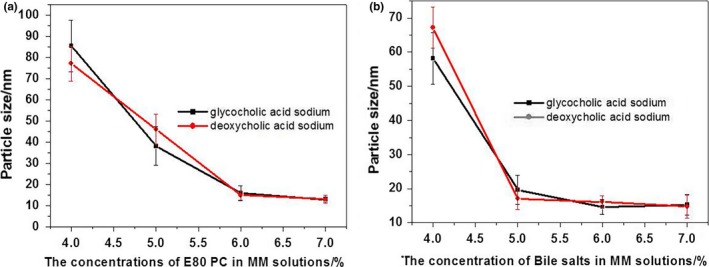
The particle sizes of mixed micelle solutions with different concentrations of phospholipids (PC) E80 and bile salts (mean ± *SD*, *n* = 3): (a) the concentrations of bile salts ranged from 2.0% to 7.0% while that of PC E80 was constant (6.0%); (b) the concentrations of PC E80 ranged from 4.0% to 7.0% while that of bile salts was constant (6.0%)

The particle sizes in MM systems were measured with a Nano ZS90 Zetasizer (Malvern Instruments). The mean sizes of MM particles rapidly decreased as the concentrations of PC or bile salts increased until a final plateau region was reached (Figure 3). In the plateau region, MM solutions with particles smaller than 15 nm were clear, which corresponds to results from the light transmittance tests. Therefore, the optimal formulation for MM systems consisted of 1% vitamin K_1_, 6% PC, and 6% bile salts.

Mixed micelle solutions containing GAS and DAS salts were prepared according to the optimal formulation described above. For pH determination, all samples were measured directly with a pH meter (Mettler Toledo). The initial pH levels of the DAS and GAS MM solutions were 8.83 and 7.57, respectively.

**Figure 4:**
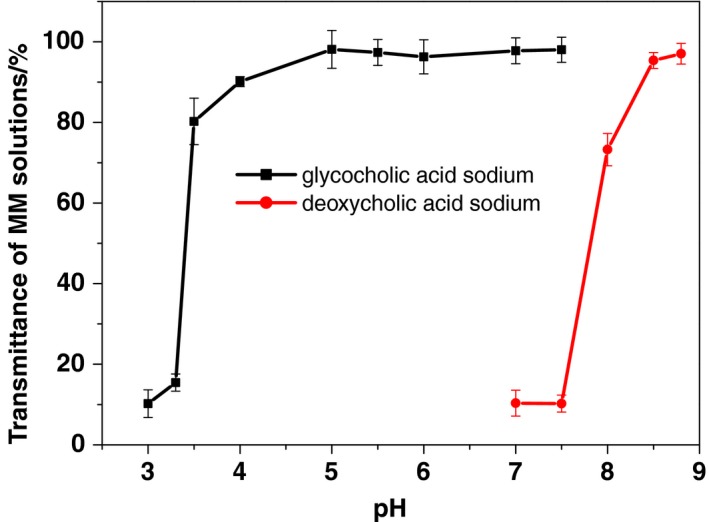
The transmittances of mixed micelle solutions after adjusting the pH from high to low values (650 nm; mean ± *SD*, *n* = 3)

**Figure 5:**
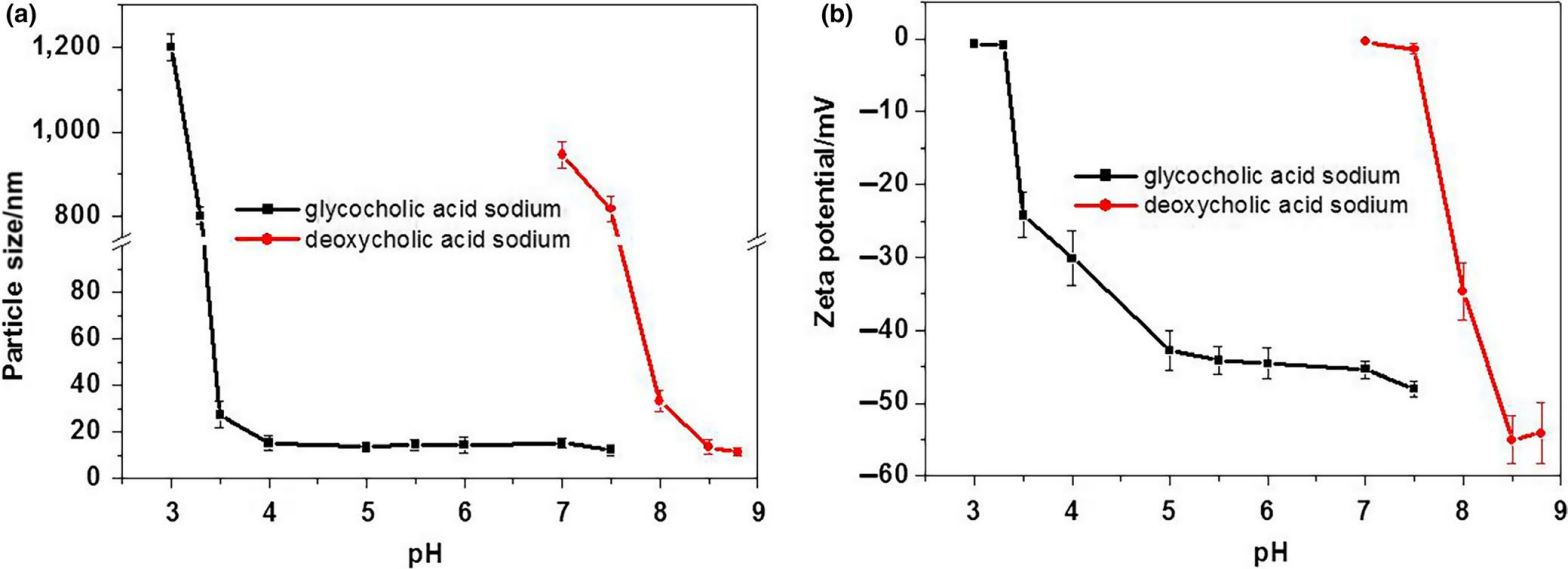
The particle sizes and zeta potentials of mixed micelle solutions after adjusting the pH from high to low values (mean ± *SD*, *n* = 3)

The GAS MM solutions were physically stable at a large range of pH levels (7.5–3.5; Figure 4). In contrast, transmittance of the DAS MM solutions quickly declined when the pH was adjusted below 8.5. The difference in pH‐related physical stability between the MM systems was related to the difference in pKa between the bile salts. The pKa of DAS (6.86) was much higher than that of GAS (3.77). Therefore, lowering the pH of the solutions affected the zeta potential of the DAS MM system more intensively than it did the GAS MM system (Figure 5). A high zeta potential indicates more electrostatic repulsion between the nanoparticles. When the zeta potential neared zero, particles of MMs precipitated and caused cloudiness.

From these experiments, the optimal pH levels for maintaining high physical stability in DAS and GAS MM systems were 8.5 and 5.5, respectively. However, more insensitive hydrolytic and oxidative degradation of active pharmaceutical ingredients was taken place in alkaline medium. Therefore, MM formulations with low pH values have higher chemical stability than those with high pH values.

**Figure 6:**
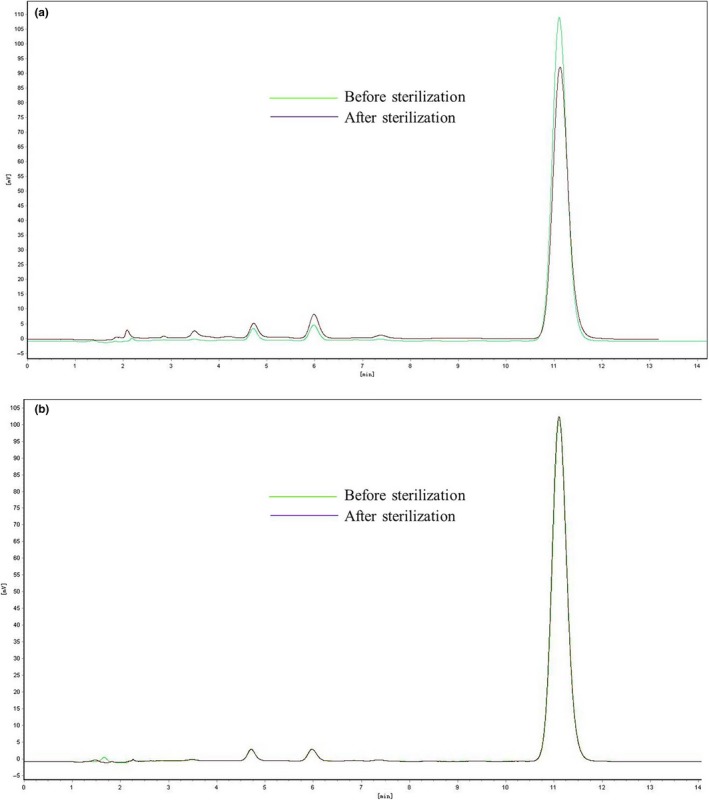
Vitamin K_1_ levels were determined by HPLC of mixed micelle solutions made with deoxycholic acid sodium (a) and glycocholic acid sodium (b)

**Figure 7:**
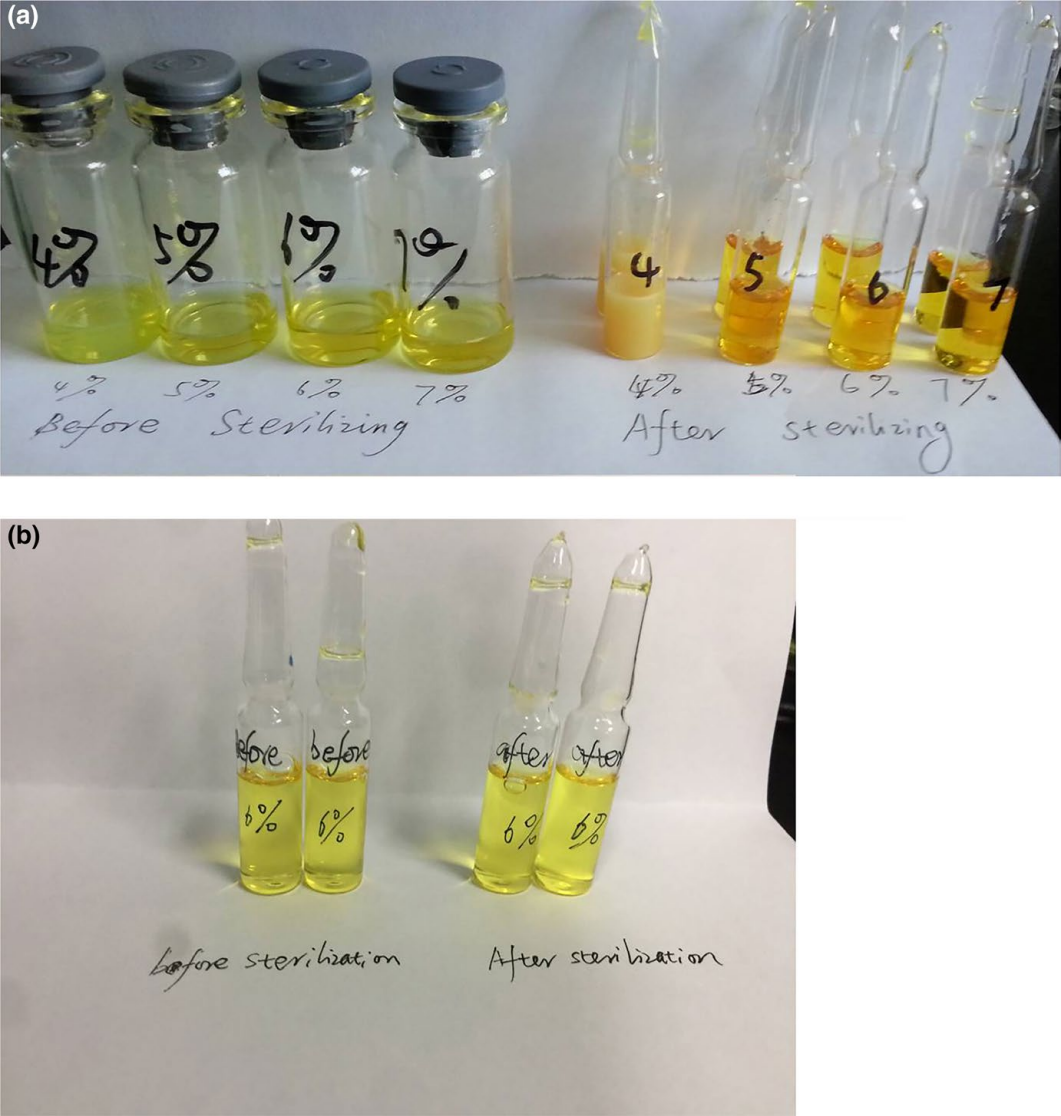
The appearance of solutions of vitamin K_1_ in mixed micelles made with deoxycholic acid sodium (a) or glycocholic acid sodium (b) before and after sterilization (121°C, 15 min)

We prepared DAS and GAS MM formulations using the methods described above, followed by adjustment of pH levels to 8.5 and 5.5, respectively. Then, they were sterilized for 15 min at 121°C. All samples were analyzed using a high‐performance liquid chromatography (HPLC) system (l‐7110/L‐7420, Hitachi) with a C18 column (Zorbax Rx‐C18, 4.6 × 250 mm, 5 μm) purchased from Agilent Instrument, Inc. The column was eluted at a flow rate of 1.0 ml/min with a 90:10 (v:v) mixture of ethanol and distilled water. The injection volume was 20 μl for samples both before and after sterilization. The detection wavelength was 215 nm, and the column was maintained at ambient temperature. For the DAS MM solutions, obvious degradation was observed following sterilization. The vitamin K_1_ content declined by 10% and the amount of impurities obviously increased. In contrast, both content and related substances in the GAS MM solutions remained unchanged after sterilization (Figure 6). Sterilization tended to improve the color change of MM solutions containing DAS rather than GAS (Figure 7), and PCs in the concentration range of 4 ~ 6% did not affect stability to sterilization. We believe that the GAS MM system did not deteriorate significantly during the sterilization process because it had a much lower pH (5.5) than that of the DAS MM system.

To summarize, low pH levels improve the stability of many active pharmaceutical ingredients, especially those that are prone to hydrolytic and oxidative degradation. Although GAS MM systems were able to maintain physical stability at low pH levels (5.5), DAS MM systems underwent considerable degradation when sterilized at their optimal pH (greater than 8.5). Thus, we conclude that DAS MM systems have limited use as parenteral drug carriers and that GAS is more suitable than DAS for the preparation of mixed micelle injections.

## CONFLICT OF INTEREST

The authors declare that there was no conflict of interests.

## ETHICAL APPROVAL

The study conforms to the Declaration of Helsinki, USA, and/or European Medicines Agency Guidelines for human subjects. The protocols and procedures in this study were ethically reviewed and approved by Ethical Committee of Beijing Institute of Pharmacology and Toxicology. This work does not involve any animal or human testing.
